# Supplementary material for: EpiMethylTag: simultaneous detection of ATAC-seq or ChIP-seq signals with DNA methylation
Source: Genome Biol. 2019 Nov 21;20:248. doi: 10.1186/s13059-019-1853-6 (PMC6868874; doi:10.1186/s13059-019-1853-6)
Supplement: Supplementary file 1 — Additional file 1: Supplementary Figure S1-S8. [file 13059_2019_1853_MOESM1_ESM.pdf]

a Number of peaks per sample

	peaks #
M-ATAC-rep1	31907
M-ATAC-rep2	30413
ATAC-rep1	28717
ATAC-rep2	37537
M-ATAC-NC-rep1	31806
M-ATAC-NC-rep2	36710
CTCF M-ChIP-rep2	33898
CTCF M-ChIP-rep1	32195
CTCF ChIP-rep1	41731
CTCF ChIP-rep2	52327
CTCF M-ChIP-NC-rep1	22576
CTCF M-ChIP-NC-rep2	31409

b Jaccard indexes of peaks intersections between samples

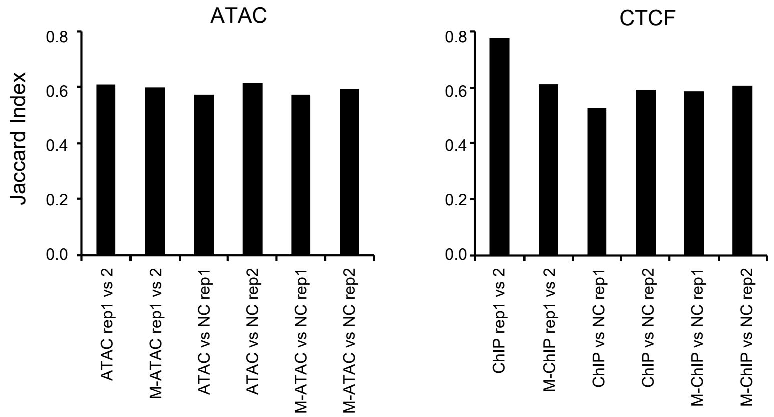

c Correlation of reads counts within peaks

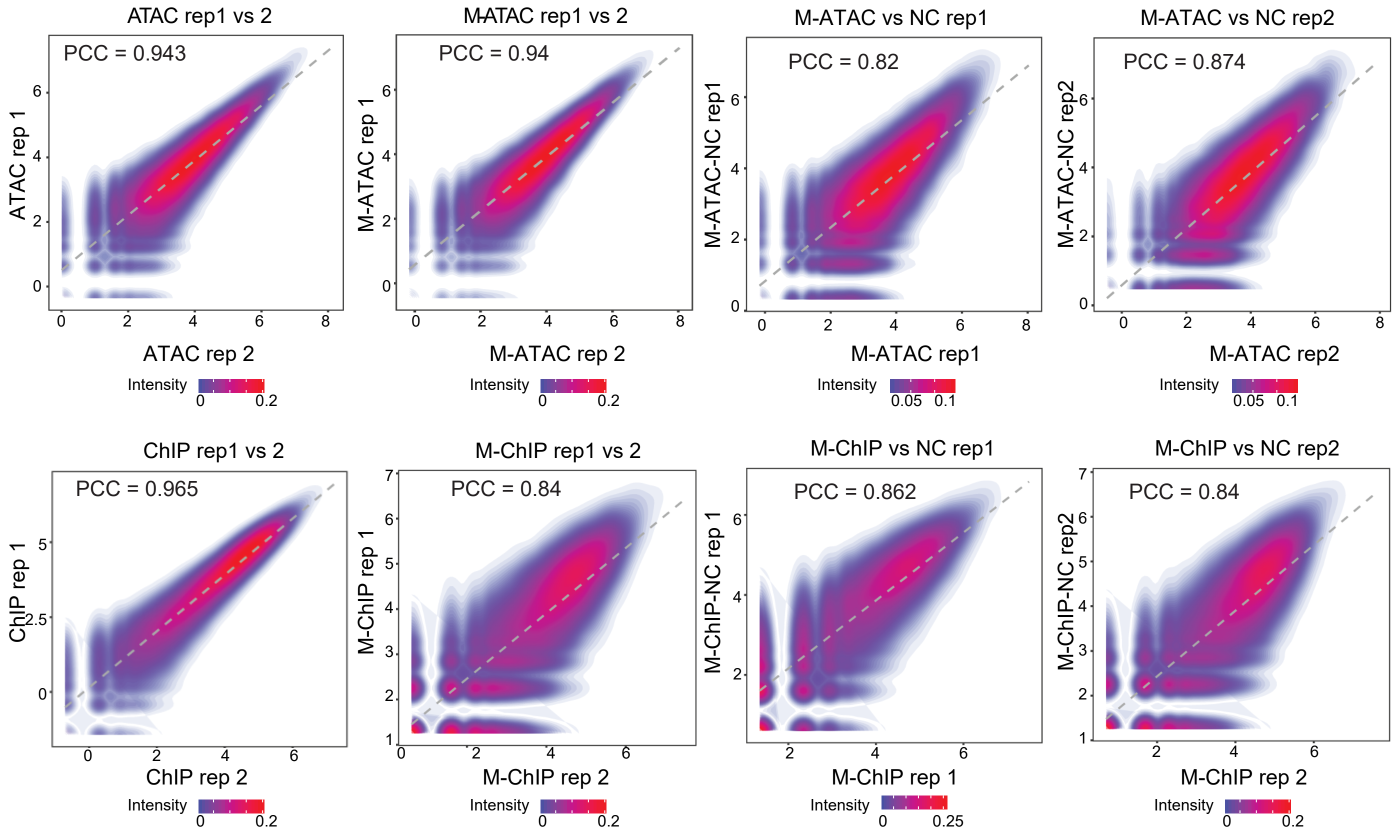

Figure S1. Peak calling in EpiMethylTag, ATAC-seq and CTCF ChIP-seq. **a** Table showing number of peaks called for each sample, using MACS2. **b** Jaccard indexes of peak intersections between ATAC, M-ATAC, M-ATAC-NC samples (left panel) and CTCF ChIP-seq, CTCF M-ChIP and CTCF M-ChIP-NC samples (right panel). Jaccard Index = (Intersection / (sample 1 + sample 2 – Intersection)). **c** Scatter plots showing correlation of signal within peaks (union of any peak found in either condition). PCC = Pearson Correlation Curve. The x and y axes represent the log2fold of read counts.

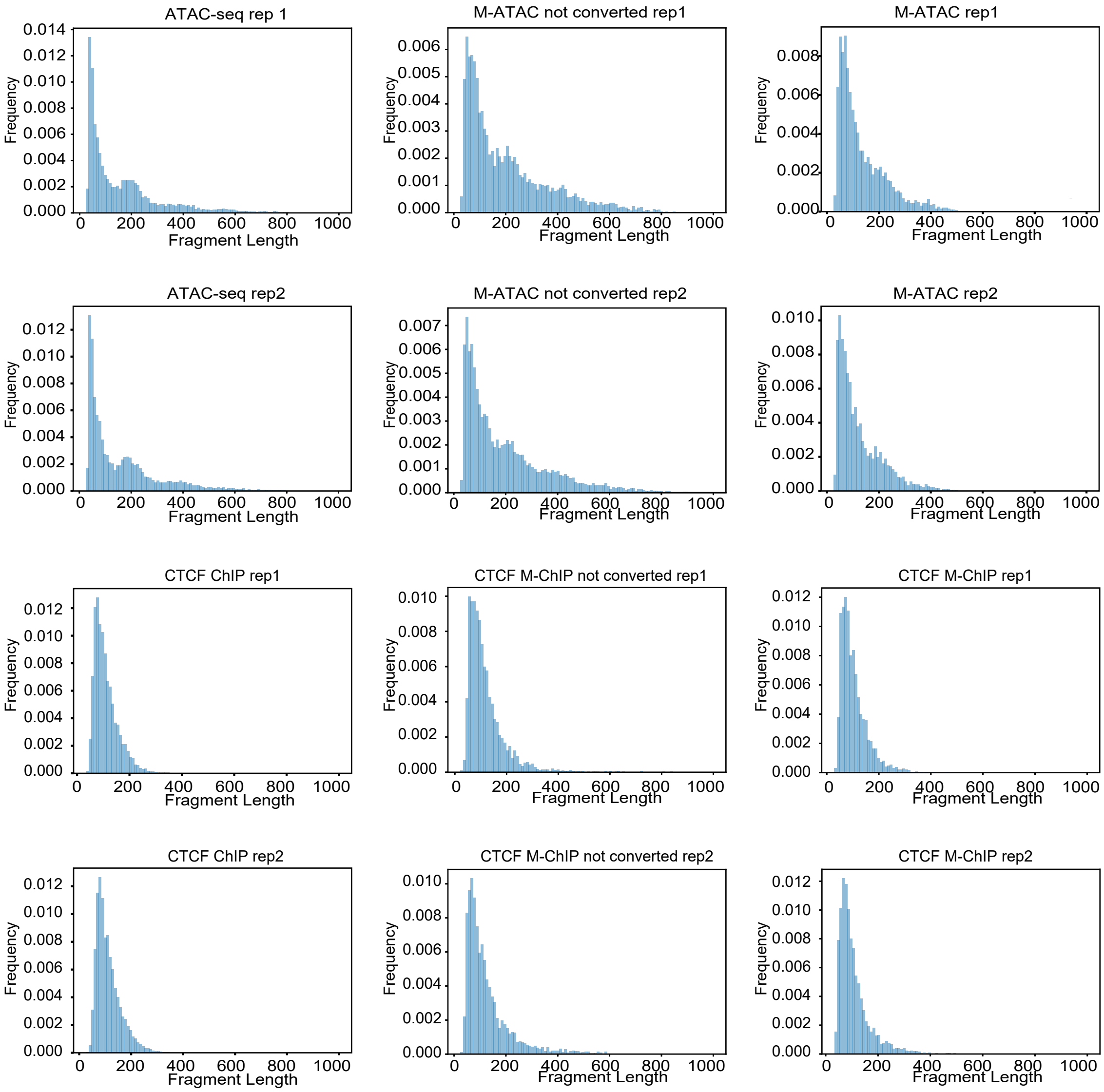

Figure S2. Read lengths for all ATAC, M-ATAC, M-ATAC unconverted (M-ATAC-NC), CTCF ChIP-seq, CTCF M-ChIP and CTCF M-ChIP unconverted (CTCF M-ChIP-NC) samples.

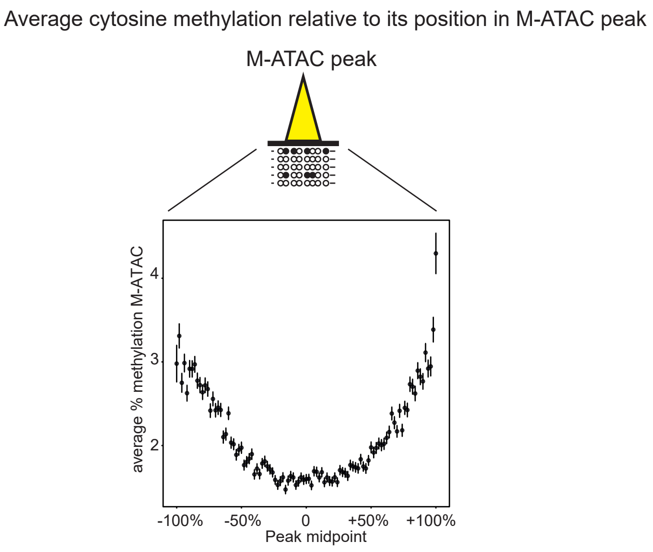

Figure S3. Average methylation in M-ATAC peaks for CpGs with coverage of at least 5 reads, relative to the position of the CpGs in the peak.

Figure S4

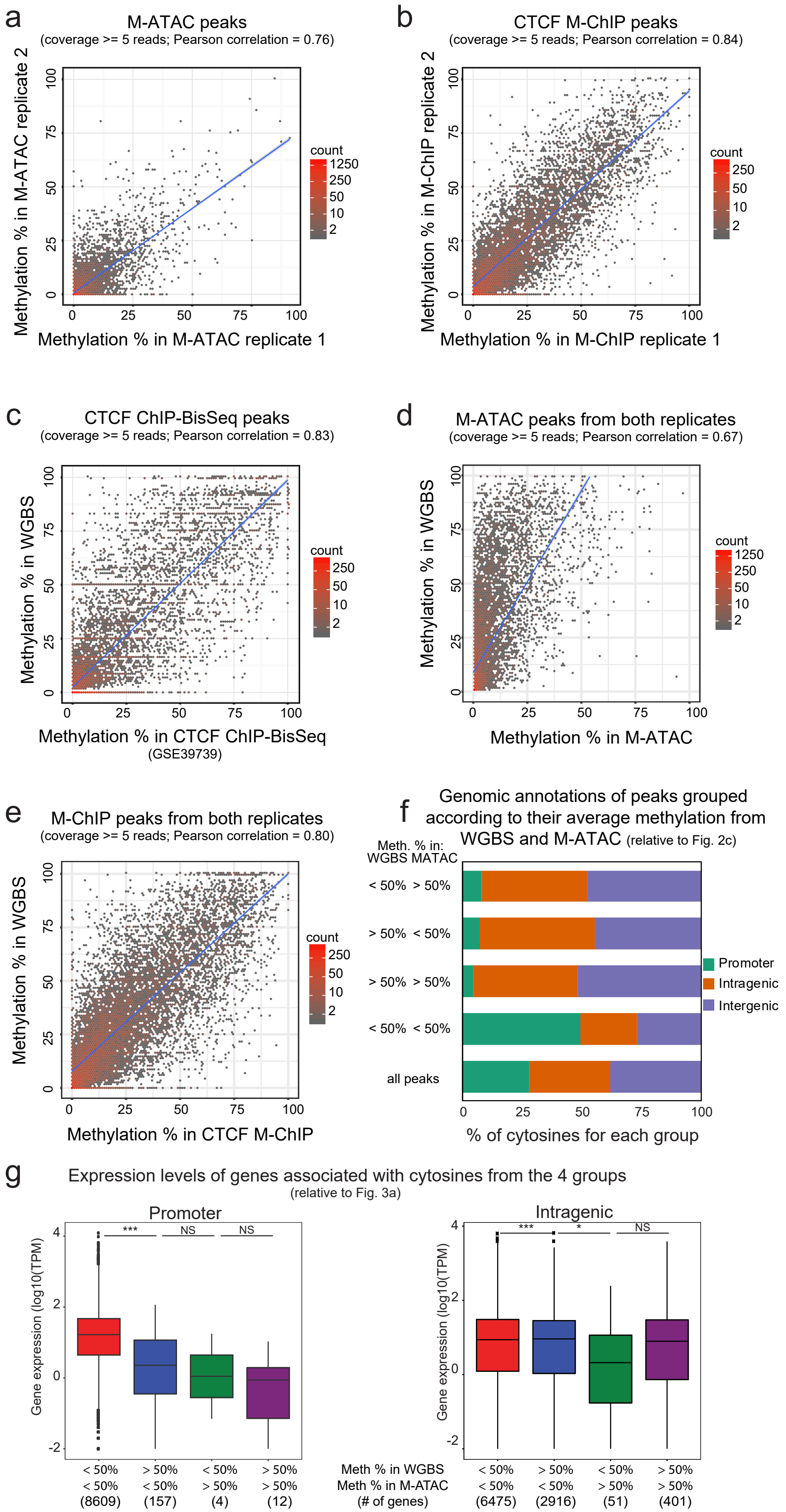
a
M-ATAC peaks
(coverage >= 5 reads; Pearson correlation = 0.76)
Methylation % in M-ATAC replicate 2
Methylation % in M-ATAC replicate 1
count
b
CTCF M-ChIP peaks
(coverage >= 5 reads; Pearson correlation = 0.84)
Methylation % in M-ChIP replicate 2
Methylation % in M-ChIP replicate 1
count
c
CTCF ChIP-BisSeq peaks
(coverage >= 5 reads; Pearson correlation = 0.83)
Methylation % in WGBS
Methylation % in CTCF ChIP-BisSeq
(GSE39739)
count
d
M-ATAC peaks from both replicates
(coverage >= 5 reads; Pearson correlation = 0.67)
Methylation % in WGBS
Methylation % in M-ATAC
count
e
M-ChIP peaks from both replicates
(coverage >= 5 reads; Pearson correlation = 0.80)
Methylation % in WGBS
Methylation % in CTCF M-ChIP
count
f
Genomic annotations of peaks grouped according to their average methylation from WGBS and M-ATAC (relative to Fig. 2c)
Meth. % in:
WGBS MATAC
< 50% > 50%
> 50% < 50%
> 50% > 50%
< 50% < 50%
all peaks
Promoter
Intragenic
Intergenic
% of cytosines for each group
g
Expression levels of genes associated with cytosines from the 4 groups
(relative to Fig. 3a)
Promoter
Gene expression (log10(TPM))
***
NS
NS
< 50%
< 50%
(8609)
> 50%
< 50%
(157)
< 50%
> 50%
(4)
> 50%
> 50%
(12)
Meth % in WGBS
Meth % in M-ATAC
(# of genes)
Intragenic
Gene expression (log10(TPM))
***
*
NS
< 50%
< 50%
(6475)
> 50%
< 50%
(2916)
< 50%
> 50%
(51)
> 50%
> 50%
(401)

Figure S4. Density plots of average methylation correlations for cytosines with coverage of at least 5 reads. **a** Average cytosine methylation from a M-ATAC replicate 1 versus replicate 2 in M-ATAC peaks (Pearson Correlation = 0.76, p-value < 2.2e-16). **b** CTCF M-ChIP replicate 1 versus replicate 2 in CTCF M-ChIP peaks (Pearson Correlation = 0.84, p-value < 2.2e-16). **c** CTCF ChIP-BisSeq (GSE39739) from Dirk Schubeler lab versus WGBS in CTCF ChIP-BisSeq peaks (Pearson Correlation = 0.83, p-value < 2.2e-16). **d** Average methylation of CpGs per M-ATAC peak in M-ATAC versus WGBS, only for M-ATAC peaks that overlap between both replicates (Pearson Correlation = 0.67, p-value < 2.2e-16). **e** Average methylation per CTCF M-ChIP peak of CpGs in CTCF M-ChIP versus WGBS only for M-ChIP peaks that overlap between both replicates (Pearson Correlation = 0.80, p-value < 2.2e-16). **f** Genomic annotations of peaks grouped according to their average methylation from WGBS and M-ATAC (relative to **Fig. 3c**, top panel). Promoter: TSS - 3kb to +3kb; intragenic: introns, exons, 5'UTR, 3'UTR and TTS, intergenic: distal from promoter >3kb. **g** Transcriptional output for the 4 groups of M-ATAC peaks according to their average methylation from WGBS and M-ATAC from **Figure S4d**, for the cytosines at promoters (left panel, see **Figure S4d**). ***P=1.25e-28 between groups #1 and 2, ^{NS}P=0.19 between groups #2 and 3, ^{NS}P=0.58 between groups #3 and 4 (Wilcoxon test), and for the cytosines at intragenic regions (right panel, introns, exons, 5'UTR, 3'UTR, see **Figure S4d**). ***P= 0.0001 between groups #1 and 2, *P= 0.02 between groups #2 and 3, ^{NS}P= 0.1 between groups #3 and 4 (Wilcoxon test).

Figure S5

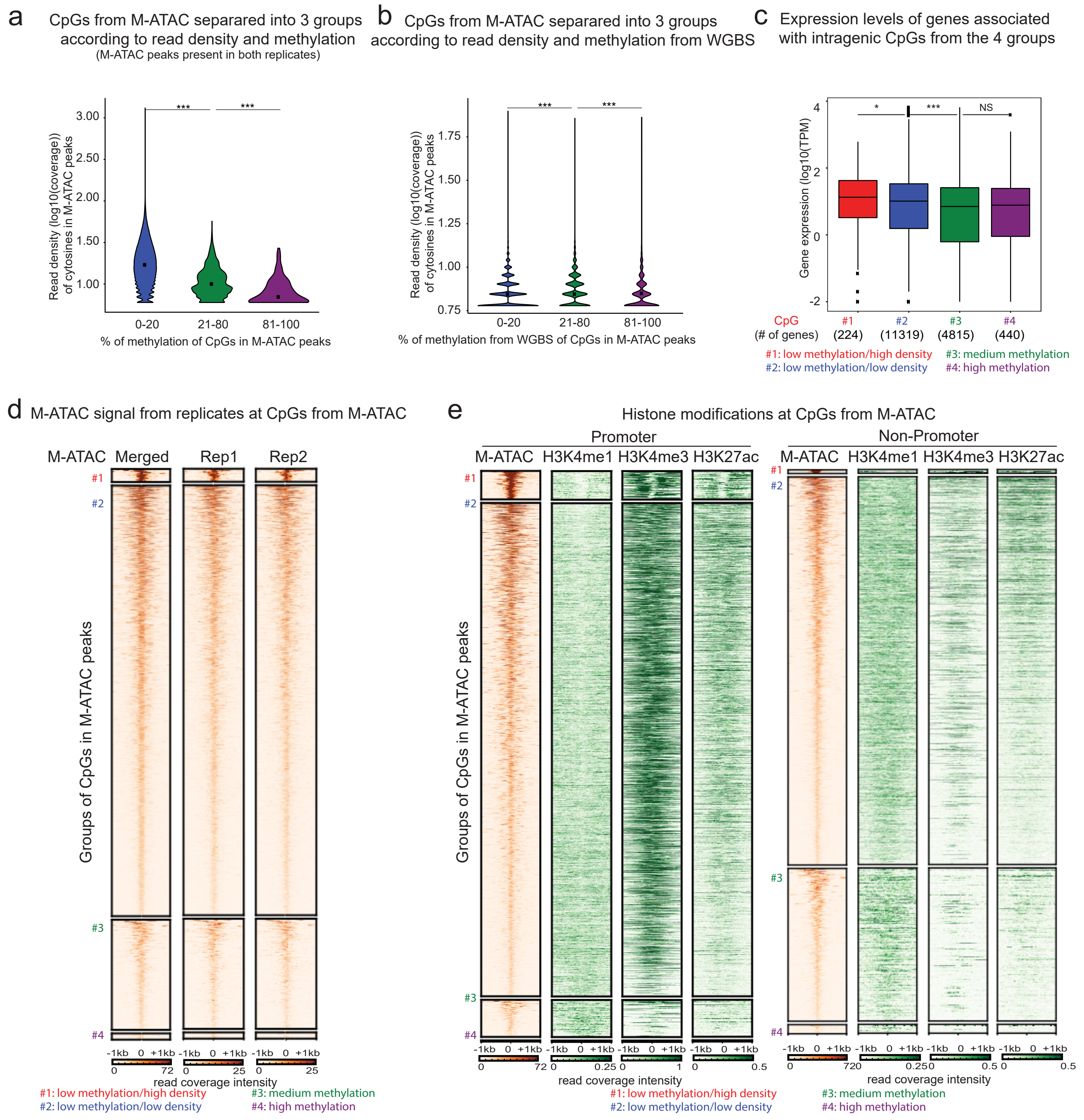

f CpGs from M-ATAC separared into 4 groups according to read density and methylation (M-ATAC peaks from merged replicates)

M-ATAC group (Fig. 3a)	1	2	3	4
number CpGs	22932	1348931	39321	1652
number M-ATAC peaks	1858	44651	17069	1137
number CTCF M-ChIP peaks	122	6526	1845	80
number CTCF motifs	206	11062	734	28
number unique CpGs within CTCF motifs (used for Fig. 4a)	288	17133	758	25
percentage of CpGs within CTCF motifs	1.26	1.27	1.93	1.51

Figure S5. M-ATAC. **a** CpGs in M-ATAC peaks that overlap between both replicates were divided into three groups according to methylation status from M-ATAC: 1/ Low Methylation (<20%, 423379 CpGs), 2/ Intermediate methylation (20-80, 7390 CpGs), 3/ High methylation (>80%, 162 CpGs). **b** CpGs in M-ATAC peaks were divided into three groups according to methylation status from WGBS: 1/ Low Methylation (<20%, 351561 CpGs), 2/ Intermediate methylation (20-80, 58655 CpGs), 3/ High methylation (>80%, 17385 CpGs). Of note, a cutoff of 5 reads coverage were applied, and as opposed to **Fig. 3a**, no additional division was made based on coverage. ***P <0.001 (Wilcoxon text). **c** Transcriptional output for the 4 groups from **Fig. 3a**, for the CpGs in M-ATAC peaks at intragenic regions (introns, exons, 5'UTR, 3'UTR, see **Fig. 3b**). *P= 0.028 between groups #1 and 2, ***P= 1.38e-38 between groups #2 and 3, ^{NS}P= 0.88 between groups #3 and 4 (Wilcoxon test). **d** Heatmaps of M-ATAC from individual replicates compared to merged replicates for the 4 groups of CpGs in M-ATAC peaks from **Fig. 3a**. **e** Heatmaps of M-ATAC, H3K4me1, H3K4me3 and H3K27ac signal corresponding to the average profiles shown in **Fig. 3d** for the 4 groups of CpGs in M-ATAC peaks from **Fig. 3a** at promoters (left panel) versus non-promoters (right panel). **f** Table showing the number of CpGs, M-ATAC and CTCF M-ChIP peaks, CTCF motifs and CpGs within CTCF motifs for the 4 groups of CpGs in M-ATAC peaks from **Fig. 3a**.

Figure S6

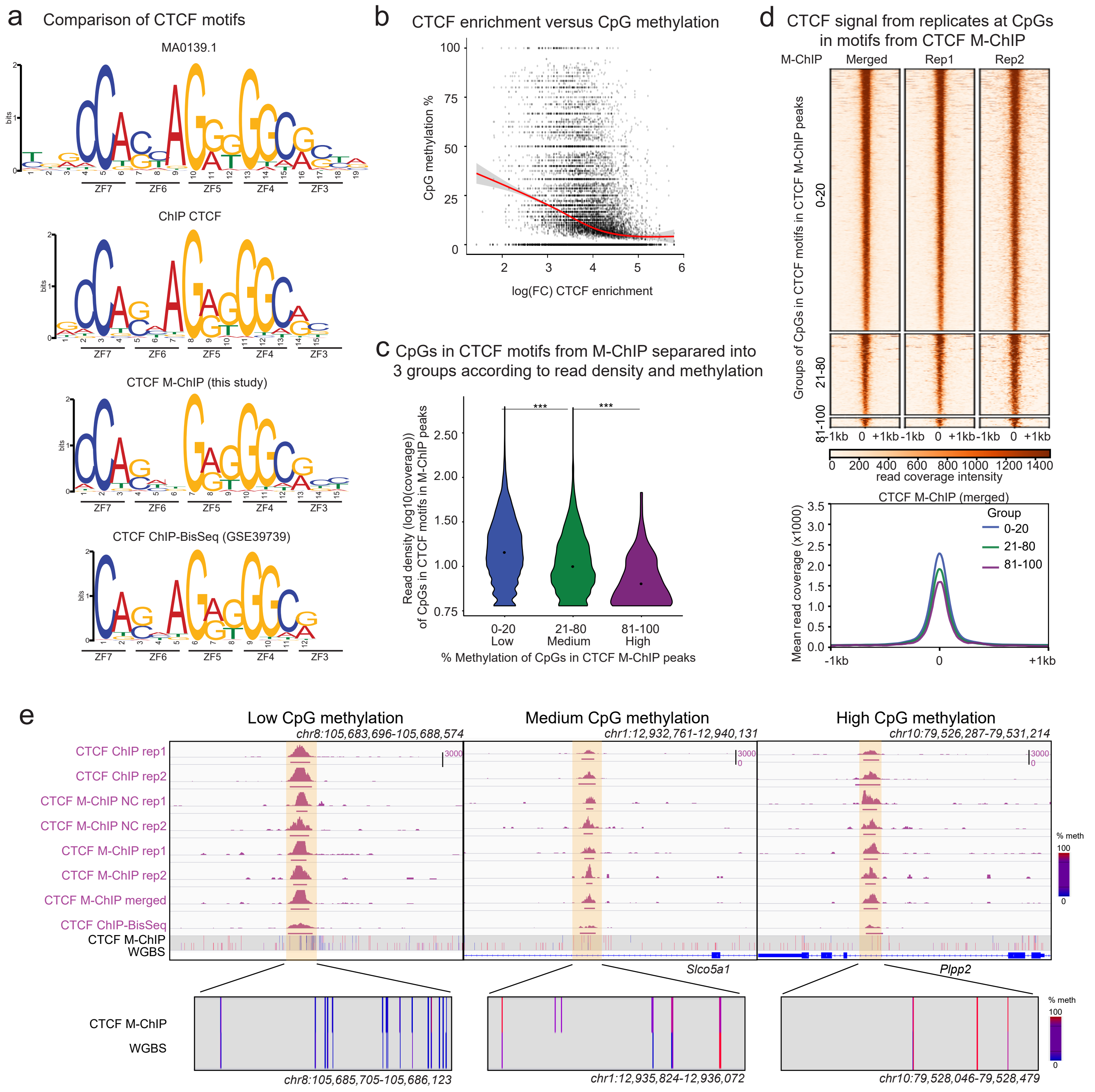

f

Number of CpGs and CTCF M-ChIP peaks in the 3 groups of CpGs based on methylation levels from M-ChIP

	CpGs within a CTCF peak			CpGs in a CTCF motif within a peak		
	0-20 Low	21-80 Medium	81-100 High	0-20 Low	21-80 Medium	81-100 High
number of CpGs (no cutoff)	103302	40389	6397	23524	4949	551
number of CpGs (cutoff 5)	81443	28793	3359	20644	3809	328
number of peaks (cutoff 5)	14460	12272	2347	8252	2551	296

g

Probability for CpGs to be in their assigned group

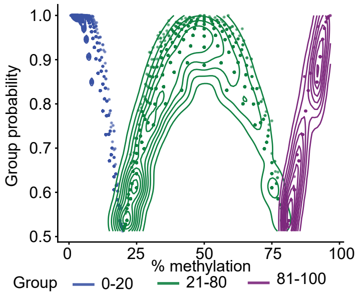

Figure S6. CTCF M-ChIP. **a** Comparison of CTCF motifs found using CTCF ChIP-seq and CTCF M-ChIP. **b** Scatter plot showing the relationship between CTCF enrichment and CpG methylation within the CTCF motifs in CTCF M-ChIP peaks. **c** CpGs within CTCF motifs in M-ChIP peaks that overlap between both replicates were divided into three groups according to methylation status from M-ChIP: 1/ Low Methylation (<20%, 20644 CpGs), 2/ Intermediate methylation (20-80, 3809 CpGs), 3/ High methylation (>80%, 328 CpGs). **d** Heatmaps of CTCF M-ChIP signal from individual replicates compared to merged replicates for the 3 groups of CpGs in M-ChIP peaks from **Figure S6c** and average profile for merged replicates. **e** Representative IGV screenshots of the 3 groups of CpGs in a CTCF motif within a CTCF peak shown in **Figure S6c** based on methylation levels from CTCF M-ChIP. **f** Table showing the number of CpGs and CTCF M-ChIP peaks depending on methylation levels of either all CpGs in CTCF peaks or only CpGs within a CTCF motif. **g** Scatter plot showing probability of the CpGs in a CTCF motif for **Figure S3c** being in their assigned group.

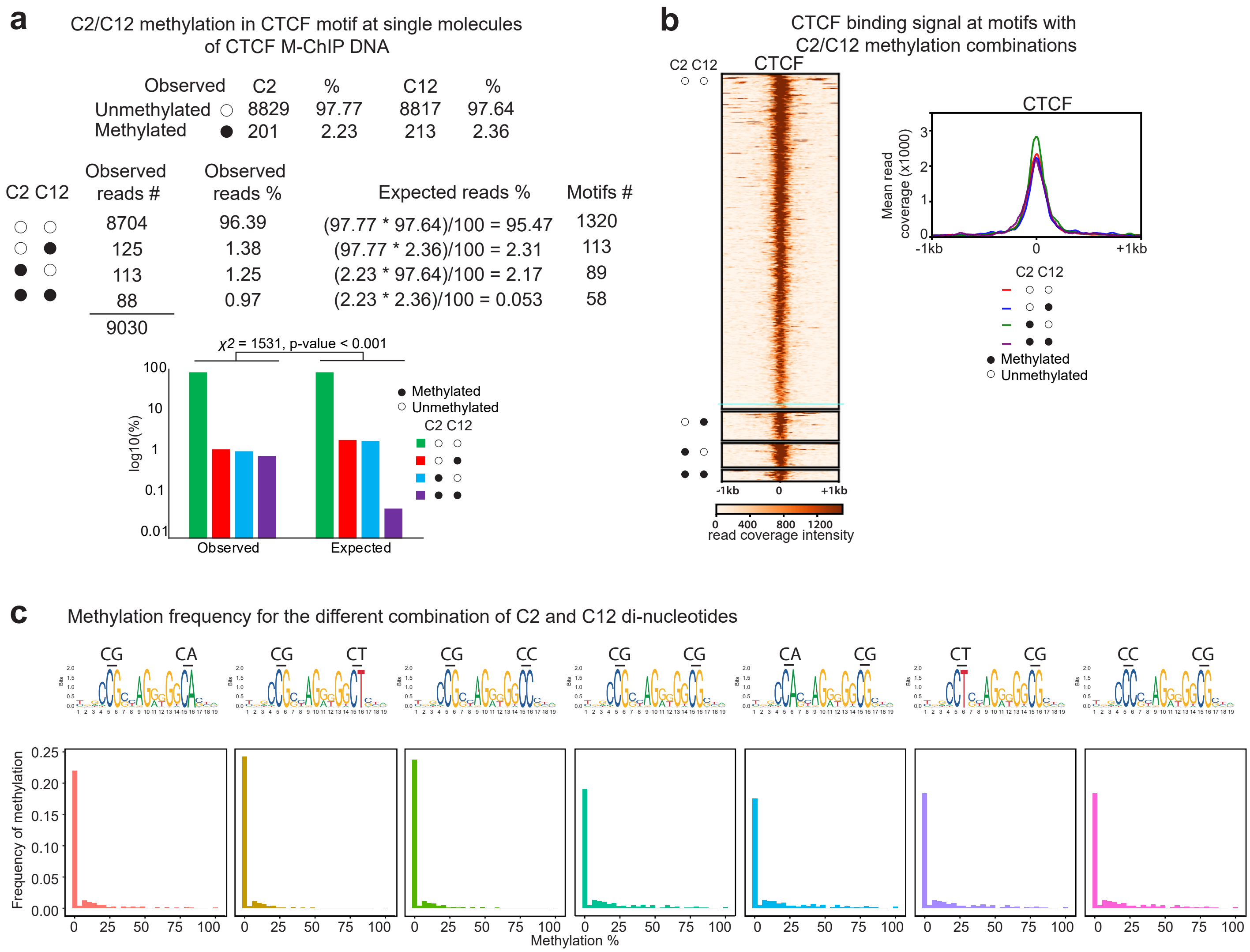

Figure S7. Methylation at C2 and C12 CpGs within CTCF motif. **a** Tables and histogram representing the number of cytosines at position C2 and C12 in the CTCF motif MA0139.1 in CTCF M-ChIP peaks as well as the frequency of the observed versus expected co-occurrence of methylation at C2 and C12 (χ2 = 1531, p- value < 0.001), and the number of CTCF motifs for each C2/C12 methylation combination. **b** Heatmap and average profile of CTCF M-ChIP signal at CTCF motifs with C2/C12 methylation combinations. **c** Frequency of methylation in the CTCF motif from CTCF M-ChIP, for the 7 possible combinations of base variations associated with C at positions 2 (1st couple of nucleotides) and 12 (2d couple of nucleotides).

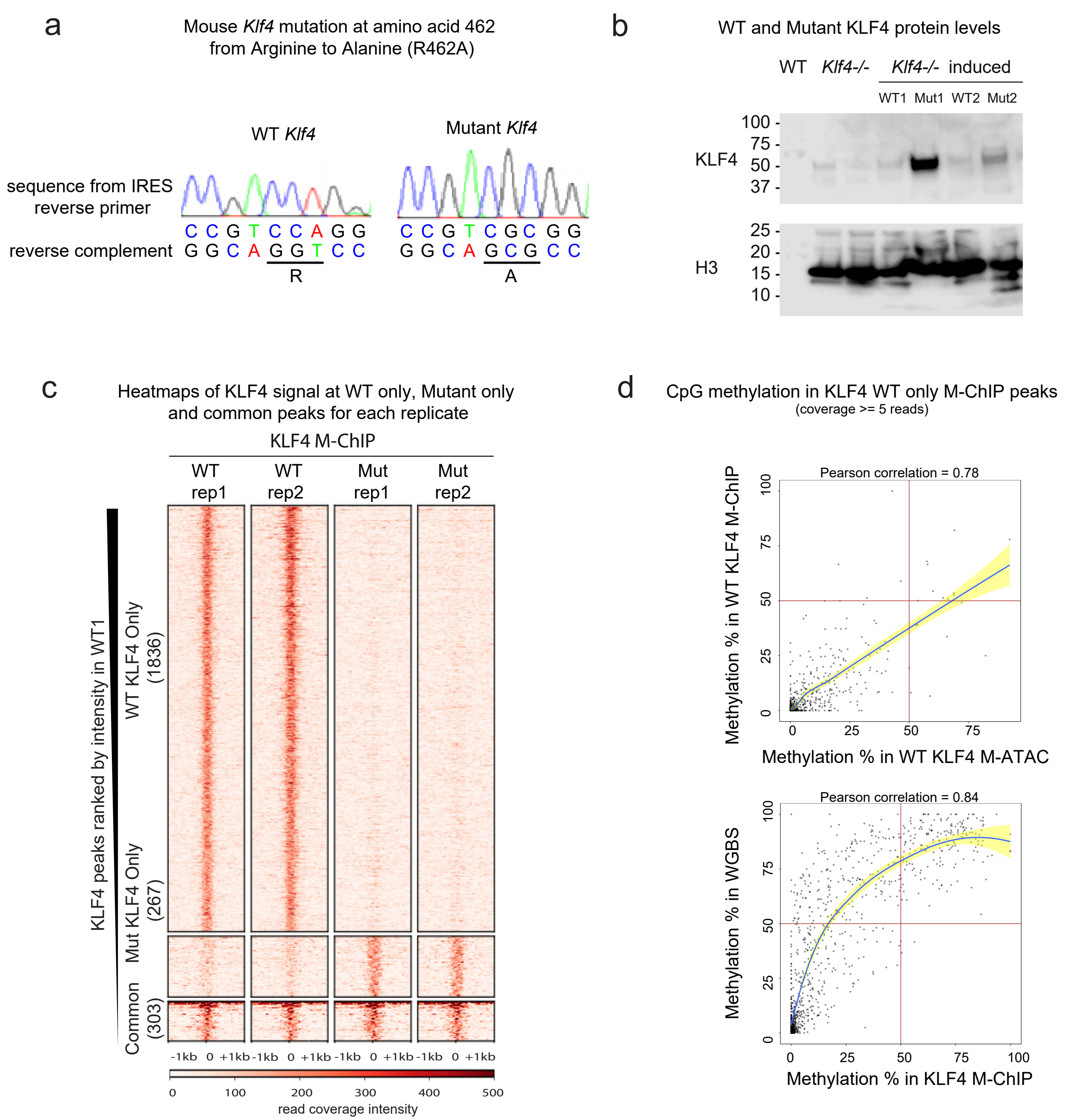

Figure S8. KLF4 mutation. **a** Sequencing from the IRES reverse primer of WT versus Mutant Klf4. The reverse complement of the sequence highlights the wild type (Arginine, R) and mutant (Alanine, A) Klf4. **b** Western blot showing the levels of KLF4 protein and H3 bulk histone in WT mESC, *Kfl4*$^{-/-}$ mESC and *Klf4*$^{-/-}$ mESC that expressed either a WT or a mutant version of KLF4. **c** Heatmap showing the binding profile of WT and mutant KLF4 R462A duplicates at WT specific (1836), mutant specific (267) and common (303) KLF4 M-ChIP peaks defined in **Fig. 4d**. **d** Average cytosine methylation from M-ATAC versus KLF4 M-ChIP in WT KLF4 expressing cells (top panel, Pearson Correlation = 0.78, p-value < 2.2e-16) and from KLF4 M-ChIP versus WGBS (bottom panel, Pearson Correlation = 0.84, p-value < 2.2e-16) for CpGs within WT specific KLF4 M-ChIP peaks.
